# Antimicrobial resistance of major clinical pathogens in South Korea, May 2016 to April 2017: first one-year report from Kor-GLASS

**DOI:** 10.2807/1560-7917.ES.2018.23.42.1800047

**Published:** 2018-10-18

**Authors:** Hyukmin Lee, Eun-Jeong Yoon, Dokyun Kim, Seok Hoon Jeong, Eun Jeong Won, Jong Hee Shin, Si Hyun Kim, Jeong Hwan Shin, Kyeong Seob Shin, Young Ah Kim, Young Uh, Ji Woo Yang, Il Hwan Kim, Chan Park, Kwang Jun Lee

**Affiliations:** 1 Department of Laboratory Medicine and Research Institute of Bacterial Resistance, Yonsei University College of Medicine, Seoul, Republic of Korea; 2 These authors contributed equally to this study; 3 Department of Laboratory Medicine, Chonnam National University School of Medicine, Gwangju, Republic of Korea; 4 Department of Clinical Laboratory Science, Semyung University, Chungbuk, Republic of Korea; 5 Department of Laboratory Medicine and Paik Institute for Clinical Research, Inje University College of Medicine, Busan, Republic of Korea; 6 Department of Laboratory Medicine, Chungbuk National University College of Medicine, Cheongju, Republic of Korea; 7 Department of Laboratory Medicine, National Health Insurance Service Ilsan Hospital, Goyang, Republic of Korea; 8 Department of Laboratory Medicine, Yonsei University Wonju College of Medicine, Wonju, Republic of Korea; 9 National Institute of Health, Centers of Disease Control and Prevention, Cheongju, Republic of Korea

**Keywords:** antimicrobial resistance, Global Antimicrobial Resistance Surveillance System, multi-drug resistance, bloodstream infection, urinary tract infection, gastroenteritis

## Abstract

The Korean government established an antimicrobial resistance (AMR) surveillance system, compatible with the Global AMR Surveillance System (GLASS): Kor-GLASS. We describe results from the first year of operation of the Kor-GLASS from May 2016 to April 2017, comprising all non-duplicated clinical isolates of major pathogens from blood*,* urine*,* faeces and urethral and cervical swabs from six sentinel hospitals. Antimicrobial susceptibility tests were carried out by disk diffusion, Etest, broth microdilution and agar dilution methods. Among 67,803 blood cultures, 3,523 target pathogens were recovered. The predominant bacterial species were *Escherichia coli* (n = 1,536), *Klebsiella pneumoniae* (n = 597) and *Staphylococcus aureus* (n = 584). From 57,477 urine cultures, 6,394 *E. coli* and 1,097 *K. pneumoniae* were recovered. Bloodstream infections in inpatients per 10,000 patient-days (10TPD) were highest for cefotaxime-resistant *E. coli* with 2.1, followed by 1.6 for meticillin-resistant *Sta. aureus*, 1.1 for imipenem-resistant *Acinetobacter baumannii*, 0.8 for cefotaxime-resistant *K. pneumoniae* and 0.4 for vancomycin-resistant *Enterococcus faecium*. Urinary tract infections in inpatients were 7.7 and 2.1 per 10TPD for cefotaxime-resistant *E. coli* and *K. pneumoniae*, respectively. Kor-GLASS generated well-curated surveillance data devoid of collection bias or isolate duplication. A bacterial bank and a database for the collections are under development.

## Introduction

Antimicrobial resistance (AMR) is a growing burden in both clinical and socioeconomic context owing to the high morbidity and prolonged hospitalisation of patients that causes elevated medical and societal costs because of loss of productivity [1]. The World Health Organization launched the Global AMR Surveillance System (GLASS) in 2015 [2] as a core global action plan addressing this issue. The standardised GLASS manual allowed an overview of global AMR rates through international comparison.

An AMR surveillance system in South Korea, the Korean AMR Monitoring System (KARMS), had been operated between 2002 and 2015 by Korean Centers for Disease Control and Prevention (KCDC) [3,4]. KARMS played an important role in notifying the high AMR rates in South Korea, urging the government to develop a national action plan. However, this system had limitations. Firstly, the antimicrobial susceptibility testing (AST) methods were not well harmonised across the participating clinical laboratories, affecting reliability. Secondly, duplicated isolates were not sufficiently filtered out, which could lead to an overestimation of the national AMR rates. Finally, the epidemiological interpretation of the study was limited because of insufficient clinical data.

From KARMS, the KCDC established an improved AMR surveillance system compatible with the GLASS, named Kor-GLASS [5]. The Kor-GLASS manual was customised from that of GLASS: (i) three bacterial species from blood specimens were added, namely *Enterococcus faecalis* and *Enterococcus faecium* to monitor vancomycin resistance and *Pseudomonas aeruginosa* to monitor carbapenem resistance and (ii) more target antimicrobial agents for AST were included to investigate multi-drug resistance by species.

We have operated the Kor-GLASS for one year since May 2016 and report here the first one-year assessment until April 2017.

## Methods

### Collection of isolates and clinical data from sentinel hospitals

The six sentinel hospitals collected bacterial isolates and clinical data, and all tests were performed in a central laboratory [5]. We collected all non-duplicated clinical isolates of *Staphylococcus aureus, Streptococcus pneumoniae, Ent. faecalis, Ent. faecium*, *Acinetobacter* spp., and *P. aeruginosa* from blood, *Escherichia coli* and *Klebsiella pneumoniae* from both blood and urine, *Salmonella* spp*.* from both blood and faeces, *Shigella* spp. from faeces and *Neisseria gonorrhoeae* from urethral and cervical swabs. Urine isolates were collected through semi-quantifying culture of urine samples following the criteria: (i) ≥10^4^ colony-forming units (CFU)/mL single-species growth of either *E. coli* or *K. pneumoniae* and (ii) ≥10^5^ CFU/mL of *E. coli* or *K. pneumoniae* in growth of mixed species [6]. We recorded epidemiological data including age, sex, infection origin (hospital origin (HO) or community origin (CO)) and admission types (outpatient department (OPD), general ward (GW), intensive care unit (ICU)) of all patients from whom blood, urine, stool or genital cultures were taken during study period. HO was defined when the specimen was taken from a patient hospitalised for two or more calendar days overall, including the hospitalisation days in another healthcare facility before transfer. CO was defined when the specimen was taken either from an outpatient or from a patient hospitalised for less than 2 calendar days.

### Microbiological analysis in the central laboratory

Bacterial species were re-checked in the central laboratory using a Bruker Biotyper (Bruker Daltonics GmbH, Bremen, Germany) and/or by nucleotide sequence analysis of the 16S rDNA or *rpoB* (for *Acinetobacter* spp.). AST was carried out by disk diffusion, Etest, and broth microdilution and agar dilution methods following the Clinical and Laboratory Standards Institute guidelines [7]. Antimicrobial susceptibility phenotypes were categorised according to Magiorakos et al. [8] with a few modifications:

• fully susceptible (DS): susceptible to all tested drugs;
• drug-resistant (DR): non-susceptible to one or two drug classes;
• multidrug-resistant (MDR): non-susceptible to three or more antimicrobial classes;
• extensively drug-resistant (XDR): susceptible to two or fewer antimicrobial classes;
• pandrug-resistant (PDR): not susceptible to any antimicrobial class.

Double-blinded inter-laboratory parallel tests were conducted monthly for randomly selected isolates in order to evaluate proficiency of the results from central and the national KCDC reference laboratories.

## Results

### Target pathogen isolation from the collected cultures

**Figure 1:**
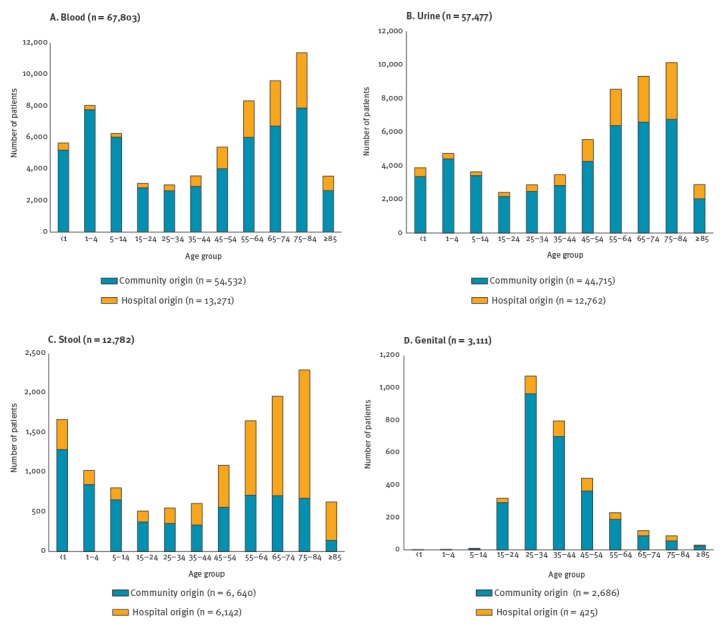
Number of patients sampled for bacterial culture, by specimen and age group, South Korea, May 2016–April 2017

During the 1-year period, six sentinel hospitals sampled 67,803 patients for blood culture, 57,477 for urine, 12,782 for stool and 3,111 for genital cultures (Figure 1). A total of 3,523 (5.2%) target pathogens were recovered from blood (Supplement: supplementary figure 1). The predominant bacterial species was *E. coli* (n = 1,536, 43.6%), followed by *K. pneumoniae* (n = 597, 16.9%) and *Sta. aureus* (n = 584, 16.6%). *Acinetobacter* spp. was recovered more often (n = 229, 6.5%) than *P. aeruginosa* (n = 127, 3.6%). The majority of *Acinetobacter* spp. were *A. baumannii* (188/229, 82.1%); the remaining 41 were non-*baumannii Acinetobacter* spp. (NBA), composed of *A. nosocomialis* (n = 23), *A. pittii* (n = 12), *A. bereziniae* (n = 2), *A. soli* (n = 2), *A. radioresistens* (n = 1) and *Acinetobacter* genomospecies 14TU (n = 1). *Ent.* *faecium* (n = 217, 6.2%) was recovered more often than *Ent. faecalis* (n = 161, 4.6%). *Salmonella* spp. (n = 44, 1.2%) and *Str. pneumoniae* (n = 28, 0.8%) were rarely recovered.

Patients are shown grouped by origin of infections. Detailed numbers can be found in Table 1 in the Supplement.

**Figure 2:**
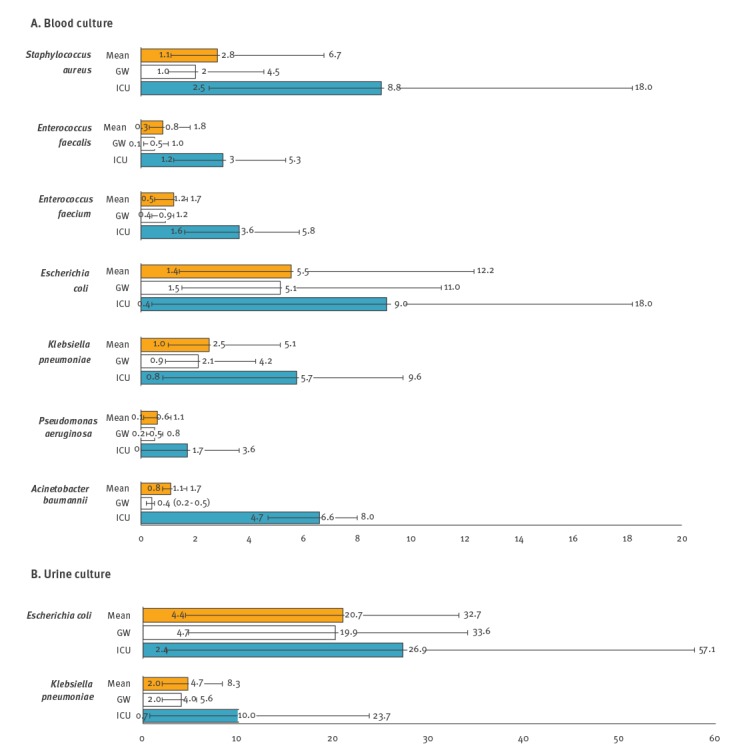
Occurrence of bloodstream and urinary tract infections per 10,000 patient-days, by target pathogen, South Korea, May 2016–April 2017 (total patient days = 1,620,431)

GW: general ward; ICU: intensive care unit.

Rates of bloodstream infection (BSI) occurrence among inpatients per 10,000 patient-days (10TPD) were calculated by bacterial pathogen (Figure 2). The total number of patient-days in the six sentinel hospitals during the surveillance period was 1,620,431 days, comprising 181,967 days in ICU and 1,438,464 days on a GW. The highest mean rate of BSI was found for *E. coli* with 5.5 (range: 1.4–12.2 by hospital), followed by 2.8 for *Sta. aureus* (range: 1.1–6.7), 2.5 for *K. pneumoniae* (range: 1.0–5.1), 1.2 for *Ent. faecium* (range: 0.5–1.7), 1.1 for *A. baumannii* (range: 0.8–1.7), 0.8 for *Ent. faecalis* (range: 0.3–1.8) and 0.6 for *P. aeruginosa* (range: 0.1–1.1). BSI occurrence was more common in ICU patients than in GW patients: the ratio of ICU:GW was highest in *A. baumannii* at 16.5 (6.6:0.4), followed by 6.0 (3.0:0.5) in *Ent. faecalis*, 4.4 (8.8:2.0) in *Sta. aureus*, 4.0 (3.6:0.9) in *Ent. faecium*, 3.4 (1.7:0.5) in *P. aeruginosa*, 2.7 (5.7:2.1) in *K. pneumoniae*, and 1.8 (9.0:5.1) in *E. coli*.

The figure shows mean occurrence across all six hospitals, with the lowest and highest indicated by error bars.

From the 57,477 urine cultures, 6,394 (11.1%) *E. coli* and 1,097 (1.9%) *K. pneumoniae* were recovered. The mean rate of *E. coli* urinary tract infection (UTI) occurrence among inpatients per 10TPD was 20.7 (range: 4.4–32.7 by hospital) and that caused by *K. pneumoniae* was 4.7 (range: 2.0–8.3). The ratio of UTI occurrence in ICU:GW was higher in *K. pneumoniae* (2.5, 10.0:4.0) than in *E. coli* (1.4, 26.9:19.9). From the 12,782 stool cultures, 77 *Salmonella* spp. (0.6%) were recovered. None of the 3,111 genital cultures were positive for *N. gonorrhoeae*.

### Antimicrobial susceptibilities of major pathogens

#### Gram-positive pathogens

**Figure 3:**
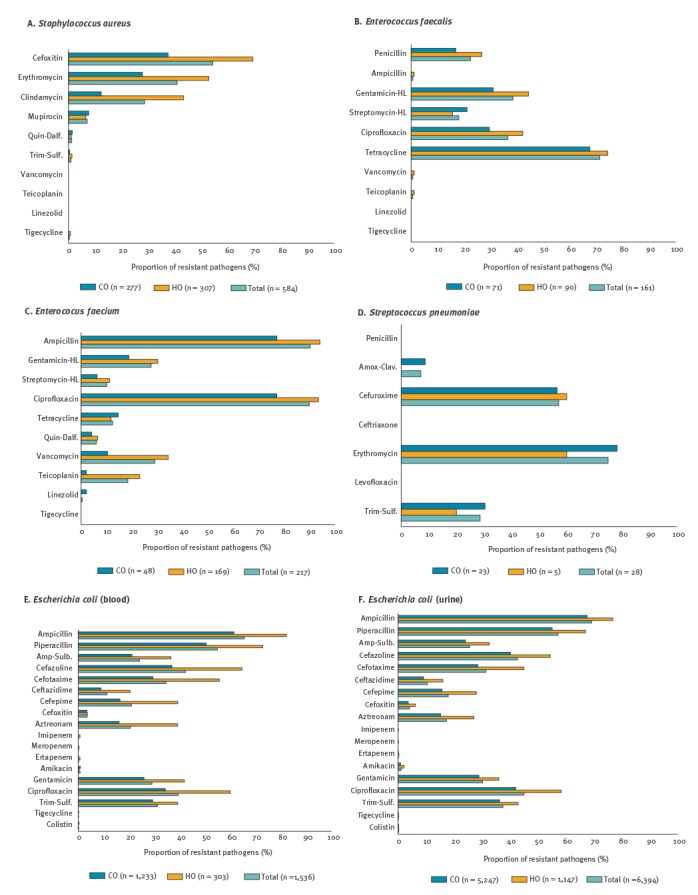
Percentage of resistance to major antimicrobials by infection origin, South Korea, May 2016–April 2017 (Part I: panels A–F)

**Figure 4:**
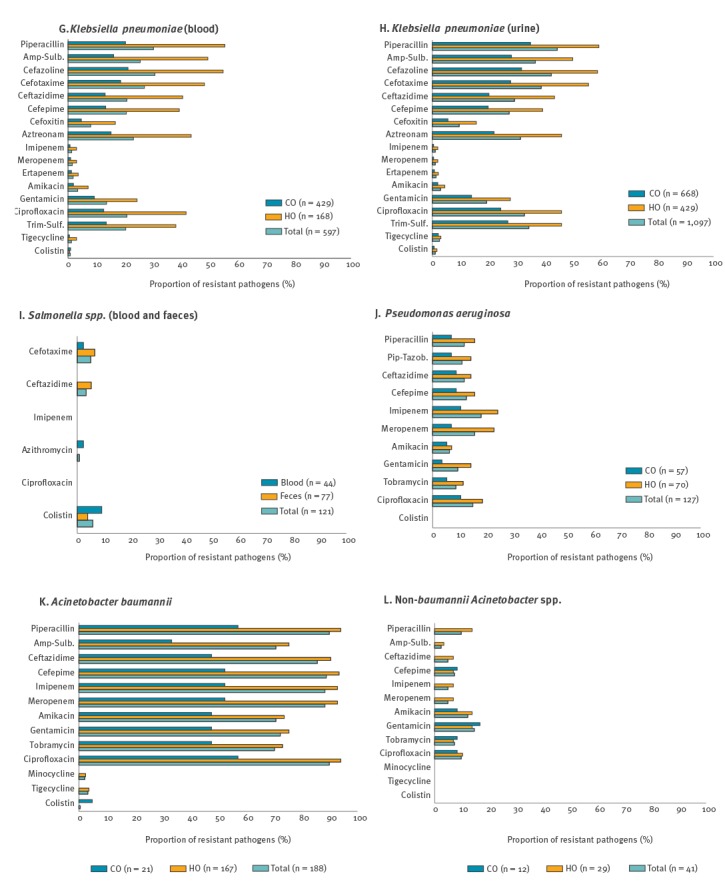
Percentage of resistance to major antimicrobials by infection origin, South Korea, May 2016–April 2017 (Part II: panels G–L)

More than half (317/584, 54.3%) of *Sta. aureus* blood isolates were resistant to cefoxitin (Figures 3 and 4), which means that these were meticillin-resistant *Sta. aureus* (MRSA). By origin of infection, 69.4% (213/307) of HO *Sta. aureus* were MRSA, more than the 37.5% (104/277) of CO. Most of the isolates remained susceptible to linezolid, tigecycline and quinupristin-dalfopristin, and they were all susceptible to vancomycin and teicoplanin. The *Sta. aureus* blood isolates were categorised as DS (35.8%, n = 209), DR (9.9%, n = 58) and MDR (54.3%, n = 317), and all MDR isolates were MRSA (Supplement: supplementary figure 2).

Amox-Clav: amoxicillin-clavulanic acid; Amp-Sulb: ampicillin-sulbactam; CO: community origin; HO: hospital origin; Gentamicin-HL: high-level resistance to gentamicin; Pio-Tazob: piperacillin-tazobactam; Quin-Dalf: quinupristin-dalfopristin; Streptomycin-HL: high-level resistance to streptomycin; Trim-Sulf: trimethoprim-sulfamethoxazole.

Detailed numbers can be found in Tables 2–7 in the Supplement.

Amox-Clav: amoxicillin-clavulanic acid; Amp-Sulb: ampicillin-sulbactam; CO: community origin; HO: hospital origin; Gentamicin-HL: high-level resistance to gentamicin; Pio-Tazob: piperacillin-tazobactam; Quin-Dalf: quinupristin-dalfopristin; Streptomycin-HL: high-level resistance to streptomycin; Trim-Sulf: trimethoprim-sulfamethoxazole.

Detailed numbers can be found in Tables 8–13 in the Supplement.

While resistance to ampicillin in *Ent. faecalis* blood isolates was rare (1/161, 0.6%), the proportion of penicillin resistance was comparably high (n = 36, 22.4%) and more frequent in HO (24/90, 26.7%) than in CO (12/71, 16.9%). Resistance to glycopeptides was also rare (Figures 3 and 4). The rate of high-level resistance [7] to aminoglycosides was 38.5% (n = 62) to gentamicin and 18.0% (n = 29) to streptomycin. The majority of the *Ent. faecalis* blood isolates were either DR (49.1%, n = 79) or MDR (42.2%, n = 68). The proportion of multidrug resistance in penicillin-resistant isolates was three times higher (86.1%, 31/36) than that in susceptible ones (29.6%, 37/125).

Among the 217 *Ent. faecium* blood isolates, 90.3% (n = 196) were resistant to ampicillin and 29.0% (n = 63) and 18.4% (n = 40) were resistant to vancomycin and teicoplanin, respectively. For these drugs, resistance rates in HO isolates were higher than those in CO isolates (Figures 3 and 4). Forty of the 63 vancomycin-resistant *Ent. faecium* (VREFM) were also resistant to teicoplanin and 14 were intermediate, while nine remained susceptible to the drug. In addition, 27.6% (n = 60) and 10.1% (n = 22) of all *Ent. faecium* were high-level resistant to gentamicin and streptomycin, respectively. Two thirds of the *Ent. faecium* blood isolates were MDR (62.7%, n = 136) and one third was DR (33.2%, n = 72). All VREFM isolates were categorised as MDR.

All *Str. pneumoniae* blood isolates were susceptible (23/28) or intermediate (n = 5) to penicillin. The five penicillin-intermediate isolates were also non-susceptible to other drugs. All 28 isolates were susceptible to levofloxacin. Erythromycin resistance was observed in 21 of the 28 isolates.

#### Enterobacteriaceae

Ampicillin resistance was identified in 65.6% (1,007/1,536) of *E. coli* blood isolates and decreased to 24.2% (n = 372) when sulbactam was supplemented. Rates of resistance to cefotaxime, ceftazidime and cefepime were 34.7% (n = 533), 11.3% (n = 173) and 21.0% (n = 322), respectively. Carbapenem-non-susceptible isolates were rarely identified. The resistance rate to ciprofloxacin was 39.5% (n = 606) and that to amikacin was low at 0.7% (n = 11). Colistin-resistant isolates were seldom identified, with 0.2% (n = 3). Resistance rates in *E. coli* urine isolates were similar to those in blood isolates. One isolate was resistant to all three carbapenems and six were non-susceptible only to ertapenem. Colistin resistance was detected in 14 (0.2%) of the 6,394 *E. coli* urine isolates. HO isolates had higher resistance rates to most of the tested antimicrobials than CO isolates, and this difference was much greater in blood isolates than in urine isolates. The vast majority (98.8%, 2,616/2,648) of cefotaxime-non-susceptible *E. coli* isolates were MDR and all XDR isolates (0.2%, 15/7,930) were non-susceptible to both carbapenems and cefotaxime.

One third (30.2%, 180/597) of *K. pneumoniae* blood isolates were piperacillin-resistant and a quarter (25.5%, n = 152) were resistant to ampicillin-sulbactam. For the extended-spectrum cephalosporins, 27.0% (n = 161) isolates were resistant to cefotaxime, 20.8% (n = 124) to ceftazidime and 20.6% (n = 123) to cefepime. Carbapenem resistance was identified more frequently in *K. pneumoniae* blood isolates than in *E. coli* (Figures 3 and 4). The resistance rate to ciprofloxacin was 20.8% (n = 124), and those to amikacin and gentamicin were 3.4% (n = 20) and 13.6% (n = 81), respectively. Colistin resistance was identified in 0.8% (n = 5) isolates. *K. pneumoniae* urine isolates exhibited higher rates of resistance to the antimicrobials tested than the blood isolates. Carbapenem resistance rates in the 1,097 urine isolates were 1.0% (n = 11) to imipenem, 1.0% (n = 11) to meropenem, and 1.3% (n = 14) to ertapenem. Colistin resistance was detected in 1.0% (n = 11) isolates. The proportion of antimicrobial resistance in *K. pneumoniae* of HO was higher than in those of CO and the difference between them was larger in blood isolates (ca. three times higher) than in urine isolates (ca. 1.5 times higher). Most of the 604 Cefotaxime-non-susceptible isolates were MDR (77.6%, n = 469) or XDR (20.9%, n = 126).

Among 44 *Salmonella* blood isolates, one isolate was resistant to cefotaxime and intermediate to ceftazidime. All the isolates remained susceptible to imipenem. None was resistant to ciprofloxacin, however, 10 were intermediate to the drug, and colistin resistance was observed in four. Five of the 77 *Salmonella* stool isolates were cefotaxime-resistant, and four of those five were also resistant to ceftazidime. Similar to the blood isolates, all stool isolates were also susceptible to imipenem and 12 isolates were intermediate to ciprofloxacin. Three isolates were resistant to colistin.

#### Glucose non-fermenting Gram-negative bacilli

Non-susceptibility to piperacillin in *P. aeruginosa* blood isolates was observed in 21 of 127 (16.5%) isolates, and supplementing tazobactam had no effect on the susceptibility proportion (82.7%, n = 105) (Figures 3 and 4). The proportion of resistance to ceftazidime and cefepime was 11.8% (n = 15) and 12.6% (n = 16), respectively. Rates of carbapenem resistance were 18.1% (n = 23) for imipenem and 15.7% (n = 20) for meropenem. In addition, 6.3% (n = 8) of isolates were resistant to amikacin, 9.4% (n = 12) to gentamicin, 8.7% (n = 11) to tobramycin and 15.0% (n = 19) to ciprofloxacin. All isolates remained susceptible to colistin. Twice as many HO than CO isolates were non-susceptible to the drug. The amikacin-non-susceptible *P. aeruginosa* blood isolates were mostly XDR (8/9) with the exception of one MDR isolate. In contrast, 14 of 30 among the imipenem-non-susceptible isolates were XDR, four were MDR and 12 were DR.

The proportion of AMR in the 188 *A. baumannii* blood isolates was higher than 70% for all tested drugs (Figures 3 and 4) and the AMR proportion in HO isolates was ca. twice that of CO isolates. Minocycline, tigecycline and colistin were still active in 93.1% (n = 175), 85.6% (n = 161) and 99.5% (n = 187) of isolates, respectively. Among the *A. baumannii* blood isolates, 76.1% (n = 143) and 13.8% (n = 26) were XDR and MDR, respectively, and all but two MDR isolates were imipenem-non-susceptible. NBA blood isolates had a markedly lower proportion of AMR than *A. baumannii*.

### The estimated prevalence of major AMR pathogens

**Figure 5:**
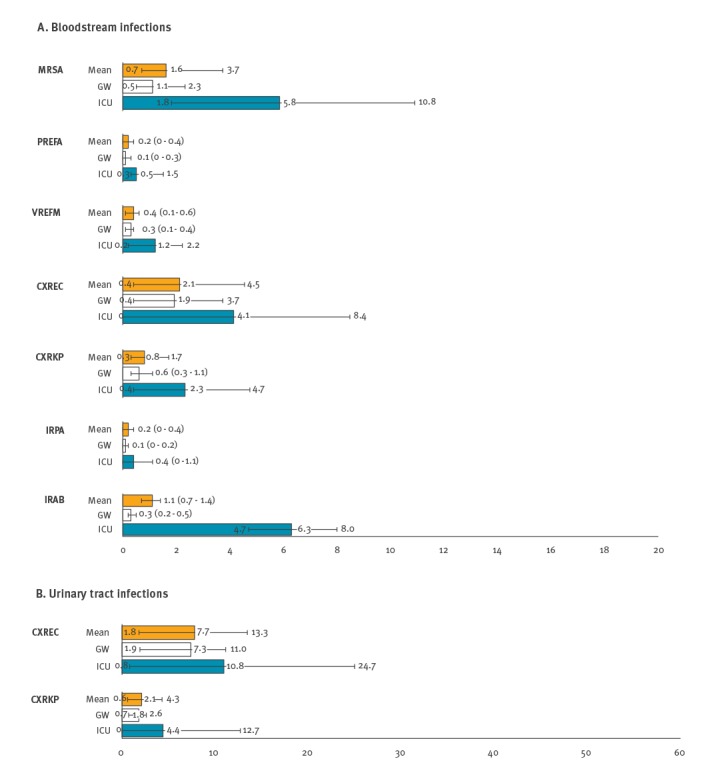
Occurrence of bloodstream and urinary tract infections per 10,000 patient-days, by major antimicrobial resistant pathogen, South Korea, May 2016–April 2017 (total patient days = 1,620,431)

CXREC: cefotaxime-resistant *Escherichia coli*; CXRKP: cefotaxime-resistant *Klebsiella pneumoniae*; GW: General ward; ICU: intensive care unit; IRAB: imipenem-resistant *Acinetobacter baumannii;* IRPA: imipenem-resistant *Pseudomonas aeruginosa*; MRSA: meticillin-resistant *Staphylococcus aureus*; PREFA: penicillin-resistant *Enterococcus faecalis*; VREFM: vancomycin-resistant *Enterococcus faecium*.

In general, the AMR proportion of major pathogens for ICU patients was higher than for GW and OPD patients (Supplement: supplementary figure 3). BSI occurrence by major AMR pathogen in inpatients per 10TPD (Figure 5) had the highest mean value of 2.1 for cefotaxime-resistant *E. coli* (CXREC), followed by 1.6 for MRSA, 1.1 for imipenem-resistant *A. baumannii*, 0.8 for cefotaxime-resistant *K. pneumoniae* (CXRKP), 0.4 for VREFM, 0.2 for penicillin-resistant *Ent. faecalis* (PREFA) and 0.2 for imipenem-resistant *P. aeruginosa*. As shown in Figure 5, the ratio of BSI occurrence of the major AMR pathogens was much higher in ICU than on GW. Mean UTI occurrence in inpatients per 10TPD was higher for CXREC at 7.7 than for CXRKP at 2.1. The ratio of UTI occurrence in ICU vs GW was higher for CXRKP at 2.4 than for CXREC at 1.5.

The figure shows mean occurrence across all six hospitals, with the lowest and highest indicated by error bars.

## Discussion

De Kraker et al. [9] described an increasing trend in the overall incidence of bacteremia caused by the top five pathogens (*E. coli*, *Sta. aureus*, *Str. pneumoniae*, *Ent. faecalis* and *Ent. faecium*) between 2002 and 2008, based on the European Antimicrobial Resistance Surveillance Network (EARS-Net; https://ecdc.europa.eu/en/about-us/networks/disease-networks-and-laboratory-networks/ears-net-about) database. Predominance of *E. coli* among pathogens causing BSIs is a common phenomenon worldwide. We assessed the relative incidence of BSIs caused by *E. coli* compared with other species: The relative ratio of *E. coli* to *Sta. aureus* in Kor-GLASS was moderate at 2.6, which was lower than the 3.2 observed in Norway [10], similar to the ratios of 2.4 in Taiwan [11] and 2.2 in Netherlands [12], and higher than the ratios of 1.2 in Japan [13], 1.2 in Vietnam [14], 1.0 in Greece [15] and 1.1 in Malawi [16]. The relative ratio of *E. coli* to *K. pneumoniae* in South Korea was also moderate at 2.6, lower than in Northern European countries (5.4 in the Netherlands and 5.0 in Norway), similar to 2.5 in Japan and 3.0 in Taiwan, and higher than 1.0 in Vietnam. *Str. pneumoniae* was frequently isolated from blood in European countries (relative ratio of *E. coli* to *Str. pneumoniae*: 3.2 in the Netherlands, 3.1 in Norway and 4.1 in Spain), but rarely identified in South Korea (ratio: 54.9) which was similar to Asian countries (ratio: 18.3 in Vietnam and 31.8 in Taiwan). The *Salmonella* spp. is still a major pathogen causing BSI in developing countries such as Vietnam (relative ratio of *E. coli* to *Salmonella* spp.: 1.4) and Malawi (ratio: 0.2), however, this species was seldom identified in South Korea (ratio: 34.9), similar to the ratios 28.1 in Spain and 14.3 in Taiwan.

*Sta. aureus* was the second most common pathogen causing BSI following *E. coli*. BSI caused by *Sta. aureus* occurred in 2.8 inpatients per 10TPD in our study, which is 1.8-fold more than the 1.6 inpatients per 10TPD measured in 2011 in a previous prospective multi-center study in South Korea [17]. Since the surveillance system of that study is not compatible to Kor-GLASS, the potential increase in *Sta. aureus*-BSI needs to be further followed up. Similarly, we observed an increased incidence of MRSA-BSI at 1.6 inpatients per 10TPD compared with 1.2 inpatients per 10TPD in 2011. This incidence was 2.5 times higher than the 0.62 inpatients per 10TBD reported in a Canadian surveillance study in 2014 [18]. The high prevalence of MRSA in blood isolates in South Korea has decreased from 72% in 2013 to 66% in 2015 as measured by KARMS [4] and further to 54.3% in 2016 according to Kor-GLASS data. EARS-Net reported similarly that the percentage of MRSA has decreased from 18.1% in 2013 to 13.7% in 2016 [9]. In addition, MRSA occurred more frequently in ICU patients than in GW patients (5.8 vs 1.1), indicating that MRSA is a problem in ICUs.

Enterococci have become a major cause of BSIs globally, owing to their intrinsic resistance to various antibiotics and their enormous ability to acquire resistance to antimicrobials. *Ent. faecium* and *Ent. faecalis* were the fourth and sixth most common pathogens causing BSI in inpatients in our study, with 1.2 and 0.8 BSI occurrences per 10TPD, respectively, and they caused over four times more BSI in ICUs than on GWs. *Ent. faecium* exhibited remarkably higher resistance rates than *Ent. faecalis* to ampicillin (90.3% vs 0.6%) and vancomycin (29.1% vs 0.6%), resulting in the identification of 1.3-fold more *Ent. faecium* than *Ent. faecalis,* similar to the 1.8-fold difference found in Spain [19]. A reversed ratio was observed in the Netherlands (0.8:1) [14], and Japan (0.7:1) [12], countries that have low rates of AMR to these drugs. The vancomycin resistance rate (29.1%) in *Ent. faecium* was higher than that of teicoplanin (18.4%) in South Korea, although all isolates were *vanA*-positive. This might be caused by the dissemination of clones with a *vanA* genotype–VanA phenotype along with a *vanA* genotype–VanD phenotype following inactivation of *vanY* and *vanZ* in the *vanA* operon by rearrangement of Tn*1546* [20]. PREFA was also a common BSI-causative AMR pathogen, especially in ICUs. Clinical impacts of the penicillin resistance need to be further investigated.

Both *E. coli* and *K. pneumoniae* exhibited higher resistance rates to cefotaxime than to ceftazidime owing to the dissemination of CTX-M-type extended-spectrum β-lactamases (ESBLs). The difference in the resistance rates to these drugs was greater in *E. coli* than in *K. pneumoniae,* which could have two possible causes: higher prevalence *K. pneumoniae* compared with *E. coli* (i) of CTX-M group 1 ESBLs, which have an expanded hydrolytic activity to ceftazidime, and (ii) of SHV-type ESBLs, which hydrolyse both cefotaxime and ceftazidime [21,22]. Identification of carbapenemase-producing Enterobacteriaceae (CPEs) in our surveillance study seemed the tip of an iceberg of the notorious AMR pathogen disseminated in clinical settings. All CPEs we identified were *K. pneumoniae* carbapenemase (KPC) producers. Continuous monitoring and action plans for CPEs are required because various types of carbapenemases, including KPC, New Delhi metallo-β-lactamase and OXA-48-likes, have been introduced to South Korea during the last decade [23-25]. Colistin resistance in *Enterobacteriaceae* was rare in our study and none contained the mobile colistin resistance (*mcr*) genes *mcr-1* to *mcr-5* [26-28], although clinical Enterobactericeae isolates carrying the *mcr-1* gene have already been reported in South Korea [29].

Amikacin non-susceptibility was a better indicator for XDR *P. aeruginosa* than imipenem-non-susceptibility. The carbapenemase-producing *P. aeruginosa* strains in South Korea harboured a class 1 integron carrying several resistance gene cassettes coding not only for a carbapenemase, but also for aminoglycoside-modifying enzymes [30]. Thus, most of the amikacin-non-susceptible isolates were XDR. However, carbapenem resistance in *P. aeruginosa* strains was conferred not only through acquisition of a gene for carbapenemase, but also through loss or alteration of the intrinsic OprD porin or overproduction of the efflux pumps [31], which rarely confer resistance to amikacin to the bacterial hosts.

BSIs caused by *A. baumannii* strains mostly occurred in ICUs rather than on GWs [32]. The likely reason is that the *A. baumannii* BSIs originated predominantly from pulmonary infections associated with ventilators. The rate of carbapenem resistance in *A. baumannii* strains was markedly high at 89.9% and most of these strains were XDR. This result is in line with previous reports from South Korea, and the resistance was mostly associated with OXA-23 carbapenemase production [33].

## Conclusion

The Kor-GLASS, which performs strain collection and centralised analysis, was launched and operated. It provided well-curated surveillance data devoid of collection bias or isolate duplication, including patient data associated with the bacterial cultures. In addition, frequency of infection occurrence by patient-days was estimated for comparison with foreign countries. The results of this surveillance helped plan national action in response to the high rate of drug resistance. A bacterial bank and a database for the collections are under development.
